# Modeling the Survival of *Escherichia coli* O157:H7 Under Hydrostatic Pressure, Process Temperature, Time and Allyl Isothiocyanate Stresses in Ground Chicken Meat

**DOI:** 10.3389/fmicb.2018.01871

**Published:** 2018-08-14

**Authors:** Chi-Yun Huang, Shiowshuh Sheen, Christopher Sommers, Lee-Yan Sheen

**Affiliations:** ^1^Institute of Food Science and Technology, National Taiwan University, Taipei, Taiwan; ^2^Eastern Regional Research Center, Agricultural Research Service, United States Department of Agriculture, Wyndmoor, PA, United States

**Keywords:** modeling, high pressure processing, allyl isothiocyanate, *E. coli* O157:H7, ground chicken meat

## Abstract

Shiga toxin-producing *Escherichia coli* O157:H7 (STEC) is a common contaminant in meat and poultry. We investigated the use of non-thermal high pressure processing (HPP), with or without allyl isothiocyanate (AITC) essential oil, to kill STEC in ground chicken meat. Temperature was found an important factor affecting the inactivation of STEC in addition to pressure and process time. A full factorial experiment design (4 factors × 2 levels) was used to facilitate and evaluate the effect of pressure (250–350 MPa), operation temperature (−15–4°C), AITC concentration (0.05–0.15%, w/w), and pressure-holding time (10–20 min) on the inactivation of STEC. A linear model (a polynomial equation) was developed to predict/describe those four parameters’ impact on *E. coli* O157:H7 survival (*R*^2^ = 0.90), as well as a dimensionless non-linear model. Both types of models were validated with data obtained from separate experimental points. The dimensionless model also demonstrated that it may predict the lethality (defined as the log CFU/g reduction of STEC before and after treatment) reasonably well with some factors set slightly outside the design ranges (e.g., a wider application than the linear model). The results provide important information regarding STEC survival as affected by HPP (e.g., pressure, time and temperature) and AITC. With the addition of AITC, the hydrostatic pressure may be lowered to the 250–350 MPa level. Regulatory agencies and food industry may use those models for STEC risk assessment in ground chicken meat. A storage test (at 4 and 10°C, 10 days) after HPP+AITC treatment indicated that AITC may continue depressing or killing the pressure-damaged cells.

## Introduction

Shiga toxin-producing *Escherichia coli* O157:H7 (STEC O157:H7) is a troublesome foodborne pathogen associated with meat contamination. Gould et al. (2013) reported the most recent and comprehensive survey of the STEC from 2000 to 2010, in which the FoodNet sites showed 2006 cases of non-O157 STEC and 5688 cases of O157 STEC infections. During 2003–2012, 390 outbreaks related to *E. coli* O157 were reported and resulted in 4,928 illnesses, in which 1,272 (26% of illnesses) hospitalizations, and 33 (0.7%) deaths. In these outbreaks, 255 outbreaks (255/390 or 65%) were caused by foods (Heiman et al., 2015). Ground meat, e.g., beef and poultry, are also among STEC reservoirs in many countries. In 2015, a STEC O157:H7 outbreak associated with chicken salad involved seven states and several illness cases was reported (Centers for Disease Control and Prevention [CDC], 2015).

In the past 10 years, high hydrostatic pressure or high pressure processing (HPP), a non-thermal technology, has continuously advanced due to better machinery design and become a commercially feasible manufacturing means to attain microbial inactivation. The pressure range, 100–800 MPa in combination with heat, the hurdle concept, has been investigated in numerous studies in recent years. HPP may enhance microbial inactivation without causing the detrimental changes to food color, flavor, nutritional content and sensory property with properly selected pressure level mostly likely at 400 MPa and lower for meat applications (Hendrickx et al., 1998; San Martín et al., 2002; Olsen et al., 2010; Buckow et al., 2013). Liu et al. (2015) reported a 1–2 log reduction of multiple verotoxigenic and non-toxigenic *E. coli* isolates suspended in ground beef treated with HPP at 600 MPa (3 min). Sheen et al. (2015) treated 39 individual STEC suspended in ground beef at 350 MPa (4°C) at multiple time points for up to 40 min using the USDA Food Safety Inspection Service (FSIS) accepted *E. coli* Petrifilms as the recovery medium to determine the D_10_ (i.e., time required to attain one log reduction) values. Ten minutes at 350 MPa produced only a one log_10_ reduction of numerous STEC, in a study that used three independent experiments for each isolate, for statistical analysis (Vaux et al., 2012). Sommers et al. (2016) determined the HPP inactivation kinetics of a multi-isolate cocktail of Uropathogenic *E. coli* (UPEC) suspended in ground chicken at 300, 400, and 500 MPa (4°C). The *D*_10_ value for HPP was 30.6, 8.36, and 4.43 min for 300, 400, and 500 MPa, respectively, using *E. coli* Petrifilms as the recovery medium. Jiang et al. (2015) obtained ca. 2–4 log reduction of STEC suspended in ground beef using 4 min × 1 min cycles of HPP followed recovery on either Tryptic Soy Agar (TSA) or Rainbow Agar O157. Bacterial injury was assessed ca. 10–40%, in good agreement with results using non-selective Aerobic Plate Count Petrifilms vs. *E. coli* Petrifilms. Baccus-Taylor et al. (2015) indicated that combining HPP (400 MPa) and temperature (30°C), the reduction of cold-shocked *E. coli* O157:H7 could only reach 1 log CFU/g. In general, HPP may deliver various pathogenic *E. coli* inactivation results in meats depending on the operation conditions and the media used to recover the survivals.

A few literatures are available with regard to application of HPP at low operation temperature (<0°C) which could affect the microbial survivals, especially for foodborne pathogens. Black et al. (2010) reported that a HPP (400 MPa, 10 min) followed by two different temperature treatments, i.e., at 20 and −5°C (freezing), the reduction of *E. coli* O157:H7 may reach 3 and 1 log CFU/g, respectively. Maresca and Ferrari (2013, 2017) reported their results on STEC and *Lactococcus lactis* spp. suspended in liquid McIlvine buffer where significant levels of inactivation could be detected at pressure > 200 MPa and temperature < −20°C. Urrutia et al. (2007) provided in-depth information and concerns for food safety, quality, process parameters and consumer acceptance with the high-pressure-low-temperature (HPLT) processing. Luscher et al. (2004) reported that water showed an “unusual” freezing depression to −22°C at 210 MPa. Three ice phases, i.e., I–III in a phase diagram of water under pressure, were presented to demonstrate the potential phase changes (solid/liquid or freezing/thawing) under high pressure. They concluded that the mechanism of *Listeria innocua* inactivation (3 log reduction at 200 MPa) in frozen suspension (buffer solution) was probably due to mechanical stress associated with phase transition. Massaux et al. (1999) studied the quality of pork meat affected by high hydrostatic pressure treatment indicating that freezing-thawing under a pressure of 100 MPa is the most interesting process for pork meat – no exudate, reducing thawing time, slight discoloration and texture toughening observed. How and what would be the survival behaviors of STEC in different foods (involving proteins, fats and other ingredients) with HPP operated at high pressure (e.g., up to 400 MPa) and low temperature (e.g., −15°C) remain to be further explored.

Phytochemicals, i.e., ascorbic acid, carotenoids, flavonoids, folic acid, and tocopherol, are naturally and widely presented in plants, fruits, vegetables, and grains. Many studies have already demonstrated the nutritional benefits of phytochemicals to include strong antioxidant, anticancer, and antibacterial properties (Zhang et al., 2003). The antibacterial properties of these compounds are due to different functional groups including alkaloids, sulfur-containing groups, terpenoids, carotenoids, and polyphenols (Dias et al., 2012; Kelley et al., 2012; Park et al., 2013; Dusane et al., 2014; Amalya and Sumathy, 2015; Dwivedi et al., 2016; Moon and Rhee, 2016). Among those, ‘wasabi,’ often used as a spice, contains a variety of phytochemicals, i.e., polyphenols, flavonoids, and allyl isothiocyanate (AITC). Previous studies have shown that AITC extracted from the dried seeds of *Brassica nigra* (black mustard) is the major antimicrobial constituent (Sultana et al., 2000; Merck, 2006). AITC also has been reported having antimicrobial (Kinae et al., 2000) and antioxidant activities (Lee et al., 2010). In addition, AITC may be used as the coating agent/film or built in packaging material to promote microbial inactivation to enhance food safety (Jin and Gurtler, 2011; Guo et al., 2013).

While an individual technology is too costly or have negative side-effects on food quality, a combination of two or more means, if properly selected, could result in a better outcome. For example, hydrostatic pressure level could be reduced by adding food-grade compounds (e.g., essential oils) to effectively enhance microbial food safety. The application of hurdle technology has become more popular in recent years to achieve multiple functions in food applications, e.g., to extend the shelf life and decrease the negative effect on food quality caused mainly by an individual technology. Somolinos et al. (2008) and Chien et al. (2017) studied the inactivation of *E. coli* by the combination of HPP and citral. Chien et al. (2016) reported the use of HPP and thymol in which STEC inactivation reached 5 log at 400 MPa, 20 min pressure time and 200 ppm of thymol. Only are a few papers available in the literature discussing the complex impact of HPP and AITC concentration on *E. coli* O157:H7 survival in foods. Li and Gänzle (2016) studied and reported the inactivation of *E. coli* with combination of AITC and pressure (450 MPa for marinated beef or 600 MPa for raw ground beef). They observed that the synergistic effect may only occur at AITC concentrations negatively effecting meat quality in beef. A storage test result at 4°C for *E. coli* was also reported.

When multiple parameters are involved in assessing a target response, mathematical modeling with proper experiment design may be the best means for the solution. Chien et al. (2016, 2017) and Sheen et al. (2018) developed linear and non-linear models to describe HPP inactivation on foodborne pathogens in combination with antimicrobial compounds and successfully demonstrated their results; therefore, similar methods were applied in this study. The objectives of this study were: (1) to evaluate the combination effects of HPP (hydrostatic pressure, operation temperature and time), and AITC concentration on the inactivation of *E. coli* O157:H7 in raw ground chicken meat; (2) to develop the four-parameter model which may properly predict the *E. coli* O157:H7 inactivation; and (3) to validate the model with experimental results.

## Materials and Methods

### Raw Ground Chicken Meat Preparation

Raw ground lean chicken meat, with no additives (95% lean, 5% fat content) purchased at a local supermarket store (Wyndmoor, PA, United States) and delivered to lab (within 1 h) in a cooler and separated into 90 ± 5 g portions in polynylon pouches (Uline, Inc., Philadelphia, PA, United States) immediately. Those samples were vacuum sealed to 50 millibars using a Multi-Vac CN200 packager (Multi-Vac Inc., Kansas City, MO, United States), then frozen (−20°C) for 24–48 h, then gamma irradiated (Cs-137, 0.070 kGy/min, −20°C, Lockheed Georgia, Marietta, GA, United States) to a dose of 10 kGy which inactivated any contaminating *E. coli* and background microflora. The irradiation-treated raw ground chicken meat showed similar lethality to the non-irradiated under HPP stress tested, e.g., the plate counts showed non-significant difference (*P* > 0.05) (Chien et al., 2016; Sheen et al., 2018). The irradiated ground chicken meat was maintained at −20°C freezer. Ground chicken meat was defrosted overnight in a refrigerator (4°C) prior to experiment procedures. Every batch of the gamma irradiated ground chicken meat was tested with TSA and non-selective petrifilm to verify that none cell count was detected.

### STEC O157:H7 Isolates and Cocktail

Three *E. coli* O157:H7 (strains: C9490, 59762 and 59768) available and purchased from the American Type Culture Collection (ATCC, Manassas, VA, United States) were used. Those isolates were individually stored at −80°C freezer. Each strain was propagated on the Sorbitol MacConkey agar at 37°C, 24 h and then stored at 4°C ready for use. One day before the experiment, a loopful of each strain was individually transferred to 10 ml Tryptic Soy Broth without glucose (TSB, BD/Difco) and held in an orbital shaker (Model G34, New Brunswick Scientific, Edison, NJ, United States) maintained at 37°C/150–180 rpm/20 h; then harvested via centrifugation at 2400 × *g*/15 min/4.0°C, (Model Z-206A, Hermle Labortechnik, Germany) and re-suspended in 20 ml of 0.1% sterile peptone water (SPW, BD/Difco) to form the three-isolate cocktail. Each culture contained 10^8–9^ CFU/ml. Fresh cocktails were prepared for each experiment.

### High Pressure Processing (HPP) Treatments

Hsu et al. (2015) described the details of HPP operation using a laboratory scale unit (Mini Food lab FPG5620, Stansted Fluid Power Ltd., Essex, United Kingdom) with a temperature control device set-up which was used in this study. When temperature below 0°C was needed, e.g., −15 to 0°C, the sample temperature was controlled by the chiller set at the targeted temperature. The tested sample temperature was further monitored by an additional T-type thermocouple (Proline RP 855, Lauda, Germany) to ensure the operation and food temperature deviation within ±1°C of set point except during the pressure come-up and release periods. All the temperature profiles showed that no thermal effects from the HPP process (i.e., <20°C in all cases). In current study with 350 MPa/10 min/-15°C conditions (for example), the initial temperature was set at −15°C. When compression started (time at 0 s), temperature rose to 10°C at 50 s (peak temperature), then gradually went down to 0°C at 130 s, continuously decreased to −13°C at 330 s and stayed steadily at −13°C until decompression began at 600 s. Temperature then dropped to −20°C for 3–5 s and rose back to −13°C (while operation completed). The temperature/time profiles (in general) were similar to that reported by Hsu et al. (2015).

### The Full Factorial Design (FFD)

To effectively investigate the four factor interactions and facilitate model development, an experimental design is needed to assist in performance of the inactivation study. Therefore, a Full Factorial Design combining four parameters was selected. Those factors included operation temperature (may involve freezing/thawing phase change in food under high pressure), hydrostatic pressure, pressure-holding time, and AITC concentration. The combinations with each factor at two levels (high and low to cover the selected range) were shown in **Table 1**. For HPP, optimal pressure was focused on 250–350 MPa, and holding times at 10–20 min. To select AITC concentration ranges, a sensory test for odor changes of ground chicken meat up to 0.15% (w/w) was conducted and found acceptable by a group of 5 lab staff. A more in-detail sensory evaluation is planned in a future study. Massaux et al. (1999) and Luscher et al. (2004) discussed and demonstrated that water freezing/thawing under high pressure may involve several ice polymorph transition and formation, its true impact on foodborne pathogen inactivation and quality damage in food matrices (e.g., meat) remains to be further investigated. They reported that inactivation of *L. innocua* was progressing rapidly during pressure holding under liquid conditions, whereas in the ice phases, extended pressure holding times had comparatively little effect. With the complexity of meat undergoing freezing/thawing at high pressure and limited information available, the phase-change (in foods) impact on a microbial inactivation (i.e., *E. coli* O157:H7) may be evaluated through the FFD data/result in which individual parameter and their interaction effects on the targeted objective (i.e., inactivation) can be estimated via a properly developed model. Modeling provides an effective tool to solve this kind of challenging problem.

**Table 1:** Factors and levels for two-level factorial design.

Factor	Levels	Low level	Middle	High level
	
	Units	−1	0	+1
Temperature	°C	−15	−5	4
Pressure	MPa	250	300	350
Time	Minute	10	15	20
AITC concentration (%)	% (w/w)	0.05	0.10	0.15

In addition to the 16 combinations (2^4^ for 4-factor × 2-level; coded +1 and −1 for high and low, respectively), a combination factors at center level (coded 0) was also selected and performed at the beginning and end of the FFD. There were total 18 sets of experiments used to develop the models. Each combination of the full factorial design was performed three times independently (Vaux et al., 2012) with two duplicated samples (3 × 2 data collected).

### Preparation of Ground Chicken Meat for HPP Treatment

Allyl isothiocyanate (≥95% FCC, Sigma-Aldrich, St. Louis, MO, United States), density 1.01 g/ml, was purchased and kept in dark/cool area. Thawed ground chicken meat (5 g) was weighed and aseptically transferred into 2 oz (Nasco Co., Fort Atkinson, WI, United States) Whirl-Pak bag/pouch; the targeted AITC concentration was pipetted and added directly into raw meat, hand-mixed for 20–30 s, then inoculated with 0.5 ml of cocktail, mixed manually for another 30 s, and sealed to 50 millibars using the Multi-Vac CN200 packager. Ten pouches were layout flat then, vacuum sealed again in a polynylon bag (Uline, Inc., Philadelphia, PA, United States) as a secondary barrier to prevent the potential contamination to HPP unit. The samples were stored at 4°C while awaiting HPP treatment, if needed due to the HPP unit capacity. The waiting time was controlled to less than 30 min. Each HPP treatment was repeated in triplicate for each of the 16 combinations in a full factorial design plus the two center points.

### *E. coli* O157:H7 Enumeration

Ground chicken meat (5 g) samples were combined with 45 ml of 0.1% SPW and stomached for 2 min (Model 400C, Seward, Basingstoke, United Kingdom). Following proper decimal dilutions with 0.1% SPW, 1.0 ml of diluted sample was placed on duplicate *E. coli*/coliform Petrifilm^TM^ (3M Microbiology Products Co., St. Paul, MN, United States). Chien et al. (2016, 2017) reported that there was not significant different in survival counts among TSA plates, *E. coli*/coliform Petrifilm^TM^ and non-selective APC Petrifilm. The films were maintained at room temperature for at least 6 h to allow the injured cells to recover (Huang, 2004), and then incubated at 37°C for 24 h. The TSA plate counts and non-selective APC petrifilm were slightly higher (ca. 0.5 log CFU/g) than those from selective 3M Petrifilm, which also has been approved/used by USDA-Food Safety Inspection Service (USDA Food Safety and Inspection Service [FSIS], 2012) for recovery of *E. coli*. However, if the actual survival count is needed for food safety consideration, the most conservative means/counts (a worst case scenario approach) may apply. Colonies were counted by the 3M Petrifilm^TM^ plate reader (Model 6499, 3M Health Care, 3M Center, St. Paul, MN, United States) and presented as log CFU/g.

### Statistical Analyses and Model Development

The results/data were analyzed by one-way analysis of variation (ANOVA) and Duncan’s multiple range tests (SAS v9.4) with *p* < 0.05 as the significance criterion. The lethality, or survival ratio, of *E. coli* O157:H7 was measured in terms of the log reduction [i.e., log (*N*_o_/*N*) = log *N*_o_ - log *N*] where *N*_o_ and *N* are the initial and survival cell counts before and after test, respectively. Statistical analysis using the general linear regression and non-linear regression procedures in SAS software (SAS v9.4, SAS Institute Inc., Cary, NC, United States) was applied to perform the data evaluation and model construction. The full factorial design is used in modeling to include terms of individual parameter, interactions of two, three, or more parameters and etc. There were many interaction terms for a four-parameter system and among those terms only significant at *P* < 0.05 were selected for inclusion. The dimensionless non-linear model, based on Sheen’s model formula, was also developed to predict the inactivation of *E. coli* O157:H7 (Zhou et al., 2015; Chien et al., 2016, 2017).

### Model Performance and Validation

Model performance may be evaluated using the experimental data vs. the predicted values. In the current case, there were 18 data points in the full factorial design which may serve for this purpose. A total 54 data set (18 × 3 duplicates) were plotted to examine how the data were bounded in the ±95% confidence interval limits.

In order to evaluate and validate the developed models, several parameter combinations within the four parameter ranges were selected to justify the linear and non-linear models. Generally speaking, the dimensionless non-linear model may cover wider parameter ranges to predict *E. coli* O157:H7 inactivation. Therefore, we also selected several parameter combinations slightly outside the factor design ranges to further evaluate the prediction accuracy of the dimensionless non-linear model.

### *E. coli* O157:H7 Storage Test

To better understand the bacterial growth potential of *E. coli* O157:H7 in poultry meat during storage (e.g., shelf life application), ground chicken meat sample pouches prepared following the same procedures mentioned before and treated with 300 MPa, 15 min at 4°C were used. After HPP, the samples were stored at 4 and 10°C for 9–10 days. The populations of *E. coli* O157:H7 in the samples were determined at selected day during the storage period (HPP treated date marked as day 1) using both selective and non-selective media as mentioned previously. The temperature at 4 or 10°C was to simulate the refrigerated storage or abuse condition, respectively. All groups were performed in triplicate randomly. The detection limit was 1.0 log CFU/g.

## Results

There were many preliminary experiments needed to determine the range of each parameter required to facilitate model development. Any single parameter having too-wide and/or too-narrow range may make the model construction task difficult or impossible. Therefore, the final selected parameter ranges may be slightly different than those individually tested. It is also interesting to note that AITC alone with concentration 0.05–0.15% showed little impact on *E. coli* O157:H7 inactivation in ground chicken meat. The performance of essential oils as inactivation enhancer may largely depend on food system and the actual impact confirmed only through experimentation.

### Physical Changes of Ground Chicken Meat – Visual Observation

The color and texture of ground chicken meat treated at 300 and 350 MPa were similar to those of untreated vacuum-packaged ground chicken meat. The raw ground chicken meat’s texture may start to deteriorate and became softer or mushy and eventually lose integrity at pressure ≥450 MPa. However, no visible color change was noticed with the addition of 0.05–0.20% AITC following a 2 weeks (4 and 10°C) storage period. Detailed experiments in texture changes will be investigated in the future.

### Hydrostatic Pressure Impact on *E. coli* O157:H7 Survival

**Table 2** shows the log reductions (lethality) of *E. coli* O157:H7, resulting from HPP (200–400 MPa, at 50 MPa intervals) at 4°C for 15 min. The *E. coli* O157:H7 reduction ranged from 0.43 (200 MPa), 0.88 (250 MPa), 1.76 log CFU/g (300 MPa), 2.10 log CFU/g (350 MPa) to 2.67 CFU/g (400 MPa). The ANOVA showed that significant dependence of reduction on MPa level (*P* < 0.05). This result further confirm that at low pressure level (e.g., 200 MPa), there is little inactivation.

**Table 2:** Logarithmic reductions of *E. coli* O157:H7 on ground chicken meat after different hydrostatic pressure treatments at 4°C and 15 min (without AITC added).

Pressur (MPa)	Inactivation Log *N*_o_ – Log *N*
400	2.67 ± 0.02
350	2.10 ± 0.08
300	1.76 ± 0.07
250	0.88 ± 0.11
200	0.43 ± 0.01

*The detection limit was 1.0 log CFU/g. Results are shown as mean ± standard deviation (N = 3 random runs; 2 duplicates/run; n = 2 × 3).*

### Allyl Isothiocyanate Impact on *E. coli* O157:H7 Survival

**Table 3** shows the log reductions of *E. coli* O157:H7 with different AITC concentration treatments (i.e., 0.05–0.25%) at 4°C and 24 h. None of the AITC concentrations showed significant effect on *E. coli* O157:H7 inactivation, where the ANOVA indicated *p* > 0.05 and all the lethality was negligible (<0.5 log CFU/g).

**Table 3:** Reductions of *Escherichia coli* O157:H7 on ground chicken meat after different AITC concentration (%) treated for 24 h.

AITC concentrate (%)	Inactivation Log *N*_o_ – Log *N*
0.05	0.26 ± 0.15
0.10	0.15 ± 0.06
0.15	0.19 ± 0.06
0.20	0.21 ± 0.05
0.25	0.35 ± 0.10

*The detection limit was 1.0 log CFU/g. Results are shown as mean ± standard deviation (N = 3, with 2 petrifilm counts per run; n = 2 × 3 random runs).*

### Hydrostatic Pressure and Temperature (−15 to 7°C) Impact on *E. coli* O157:H7 Survival

**FIGURE 1:**
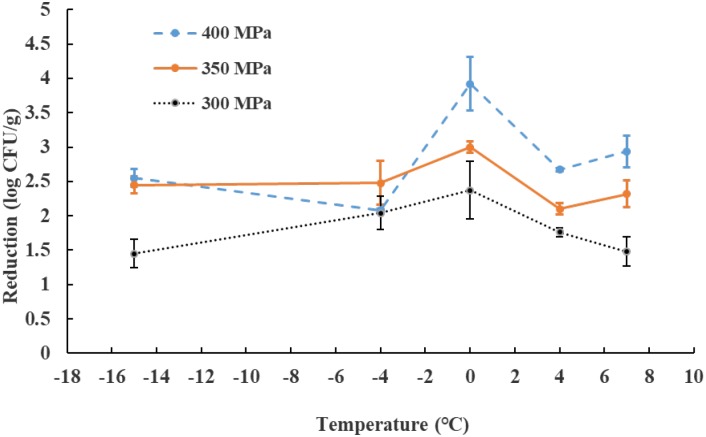
Logarithmic reductions of *Escherichia coli* O157:H7 on ground chicken meat after different high pressure level treatments at different operation temperatures for 15 min. The initial inoculum count of the *E. coli* O157:H7 was at 8.0 log CFU/g level. The detection limit was 1.0 log CFU/g. Results are shown as mean ± standard deviation (*N* = 3, with 2 petrifilm counts per run; *n* = 2 × 3 random runs).

**Figure 1** shows the log reduction of *E. coli* O157: H7, resulting from the treatment with three different pressures (300, 350, and 400 MPa) and five temperatures (−15, −4, 0, 4, and 7°C) in 15 min. The reduction of *E. coli* O157:H7 at −15, −4, 0, 4, and 7°C operation temperature was found 1.45–2.55, 2.04–2.48, 2.37–3.92, 1.76–2.67, and 1.48–2.94 log CFU/g, respectively. It was also observed that the reduction of *E. coli* O157:H7 at 0°C is higher than the other temperatures tested at 350 and 400 MPa. However, at 300 MPa level, the difference could become less significant. The selected temperature range certainly induces multiple phase changes – freezing and thawing before and after temperature treatment at −15°C; thawing and freezing during compression and decompression, respectively. The phase-change under high pressure influenced further complicates the evaluation of temperature impact on microbial survival. The −15 and 4°C were selected as the low and high end points to take advantage of treated products may be transferred to freezer and refrigerated storage, respectively, without further temperature treatment.

### HPP (Pressure, Temperature, and Time) and AITC Impact on *E. coli* O157:H7 Survival

In order to effectively evaluate the four parameters, i.e., hydrostatic pressure, operation temperature, pressure holding time and AITC concentration, impact on *E. coli* O157:H7 survival, a 2-level FFD was performed, i.e., **Table 4** where −1 and +1 indicate the lower and upper parameter level, respectively. No. 1 and No. 18 were added with all parameters at mid-level in the model development process which may enhance model prediction performance. With HPP initially set at 300–400 MPa range, the 400 MPa combination constantly showed an overwhelming reduction result. Therefore, the pressure range was adjusted down to 250–350 MPa then it was possible to implement and complete the model development.

**Table 4:** Logarithmic reductions of *E. coli* O157:H7 on ground chicken meat after high pressure processing treatments according to the four-parameter, two-level factorial design.

Trail no.	Temperature celsius (level)	Pressure MPa (level)	Time minute (level)	AITC concentration (%) % (w/w) (level)	Inactivation Log *N*_o_ – Log *N*
					*E. coli* O157:H7
1	−5 (0)	300 (0)	15 (0)	0.10 (0)	3.82 ± 0.31
2	−15 (−1)	250 (−1)	10 (−1)	0.05 (−1)	1.34 ± 0.08
3	4 (+1)	250 (−1)	10 (−1)	0.05 (−1)	0.85 ± 0.08
4	−15 (−1)	350 (+1)	10 (−1)	0.05 (−1)	2.72 ± 0.29
5	4 (+1)	350 (+1)	10 (−1)	0.05 (−1)	2.26 ± 0.11
6	−15 (−1)	250 (−1)	20 (+1)	0.05 (−1)	2.19 ± 0.1
7	4 (+1)	250 (−1)	20 (+1)	0.05 (−1)	1.60 ± 0.02
8	−15 (−1)	350 (+1)	20 (+1)	0.05 (−1)	6.38 ± 0.26
9	4 (+1)	350 (+1)	20 (+1)	0.05 (−1)	2.88 ± 0.12
10	−15 (−1)	250 (−1)	10 (−1)	0.15 (+1)	2.43 ± 0.21
11	4 (+1)	250 (−1)	10 (−1)	0.15 (+1)	2.00 ± 0.17
12	−15 (−1)	350 (+1)	10 (−1)	0.15 (+1)	5.79 ± 0.05
13	4 (+1)	350 (+1)	10 (−1)	0.15 (+1)	6.70 ± 0.83
14	−15 (−1)	250 (−1)	20 (+1)	0.15 (+1)	5.41 ± 0.42
15	4 (+1)	250 (−1)	20 (+1)	0.15 (+1)	5.85 ± 0.67
16	−15 (−1)	350 (+1)	20 (+1)	0.15 (+1)	7.18 ± 0.04
17	4 (+1)	350 (+1)	20 (+1)	0.15 (+1)	7.25 ± 0.09
18	−5 (0)	300 (0)	15 (0)	0.10 (0)	3.20 ± 0.15

*(16 design combinations + 2 center points at nos. 1 and 18). The initial inoculum count of the E. coli O157:H7 was 8.10 log CFU/g. The detection limit was 1.0 log CFU/g. Results are shown as mean ± standard deviation (N = 3 random runs; with 2 duplicates/run; n = 2 × 3 data per combination).*

The reduction of *E. coli* O157:H7 ranged from 0.85 to 7.25 log CFU/g in the full factorial design. When hydrostatic pressure level, AITC concentration and pressure-holding time increased, the *E. coli* O157:H7 survival decreased.

### Linear Model for *E. coli* O157:H7 Survival

General Regression Analysis (forward or backward stepwise) of a 4-factor, 2-level full factorial experimental design may generate the following equation for the pathogen inactivation calculation (cell count reduction).

(1) $$\begin{array}{lll} Log(N_{o}/N) & = & Y=B_{0}+B_{1}\cdot P+B_{2}\cdot C+B_{3}\cdot t+B_{4}\cdot T\\ & & +B_{5}\cdot P\cdot C+B_{6}\cdot P\cdot t+B_{7}\cdot P\cdot T+B_{8}\cdot C\cdot t\\ & & +B_{9}\cdot C\cdot T+B_{10}\cdot t\cdot T+B_{11}\cdot P^{2}+B_{12}\cdot C^{2}\\ & & +B_{13}\cdot t^{2}+B_{14}\cdot T^{2}+\cdot \cdot \cdot \cdot +B_{15}\cdot P^{3}+Bn\cdot T^{3} \end{array}$$

In Eq. (1), *Y* is the dependent target function or dependent variable. *B*_i(0−N)_ is the regression constant for each corresponding term to be determined via regression procedures. The polynomial linear model therefore, developed to predict the *E. coli* O157:H7 inactivation amount/quantity is shown in the following equation (2) using the PROC GLM procedure (SAS v9.4).

*E. coli* O157: H7 reduction (*Y* = Log *N*_o_/*N*):

(2) $$\begin{array}{lll} Y & = & 6.19509-0.07290\cdot P-0.81711\cdot C+0.25242\cdot t\\ & + & 0.03140\cdot T+0.07450\cdot P\cdot C-0.00055\cdot P\cdot t\\ & - & 0.00025\cdot P\cdot T+0.72167\cdot C\cdot t+0.79386\cdot C\cdot T\\ & & -\;0.00411\cdot t\cdot T+0.00016.P^{2} \end{array}$$

*R*^2^ = 0.90

Where, *Y*: log cell reduction in terms of the log CFU/g difference at before and after stress treatment presented as lethality per our definition in the Abstract; *P*: Pressure (MPa); *C*: AITC concentration (%, weight basis); *t*: pressure-holding time (minute); *T*: temperature (°C). The second order of *C*, *t*, and *T* terms, cubic and higher order terms were found not significant (*P* >> 0.05) in general regression analyses (GLM, SAS), therefore, those terms are all excluded. The regression analysis further confirmed that operation temperature is a significant factor in Eq. (2) where the *T* term having *p* = 0.0233 and interaction terms, i.e., *P*⋅*T*, *C*⋅*T, t*⋅*T*, all showing *p* < 0.05.

### Non-linear Model for *E. coli* O157: H7 Survival

According to Zhou et al. (2015), Sheen’s dimensionless non-linear model is a useful application to simplify a model which may involve multiple parameters. Using the non-linear regression procedure in SAS v9.4, the model developed is shown in the following equation (3):

*E. coli* O157: H7 reduction (*Z* = Log *N*_o_/*N*):

(3) $$\begin{array}{l} Z=29.5243\cdot {\left[\frac{P-200}{P+200}\right]}^{0.6417}\cdot {\left[\frac{C-0.04}{C+0.04}\right]}^{0.4005}\\ \;\cdot {\left[\frac{t-5.0}{t+5.0}\right]}^{0.6544}\cdot {\left[\frac{20-T}{20+T}\right]}^{0.0441} \end{array}$$

*F*-value = 159.72; Pr > *F*(<0.0001);

Sum of Squares Error and Sum of Squares Uncorrected Total are 61.2894 and 1060.2, respectively.

**FIGURE 2:**
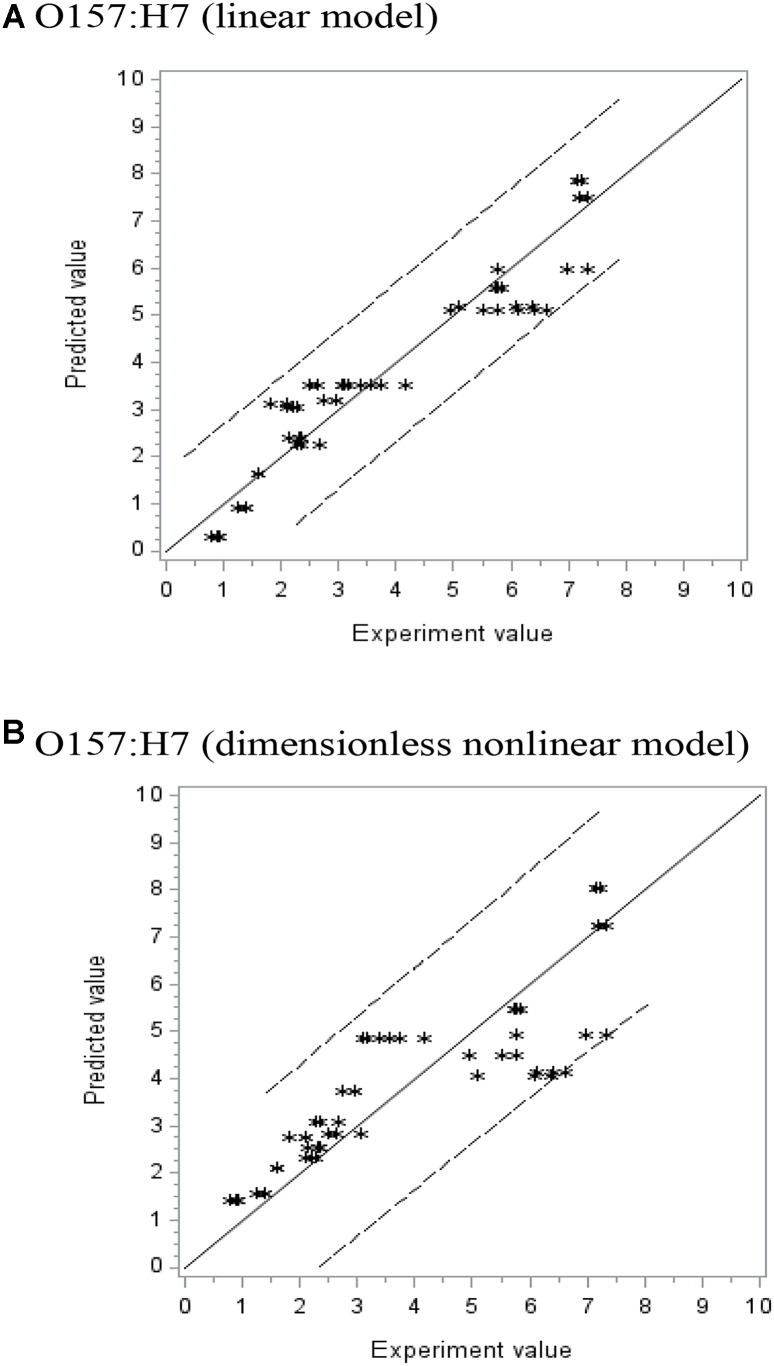
The experiment vs. predicted lethality (log CFU/g reduction) using **(A)** polynomial linear and **(B)** dimensionless non-linear models (Sheen’s model).

Where, 200, 0.04, 5.0, and 20 are the pressure *P*, AITC concentration *C*, pressure-holding time *t*, and temperature *T*, respectively, selected from experiment observations to represent a potential maximum or minimum limit of a parameter. The *F*-values and Pr > *F*-values (used in non-linear regression) indicated the equation is a good fit of the data set. **Figure 2** shows the observed experimental values versus predicted ones for the polynomial linear model (A) and Sheen’s dimensionless non-linear model (B). The solid line (slope = 1) indicates where the predicted data match the observed experimental ones. If the predicted data are over- or underestimated, the data points should be above or below the solid line, respectively. From **Figure 2**, both types of models showed good fit within the 95% confidence limits. Since the 95% confidence interval in (B) is slightly wider than (A), it is expected that predicted values using the linear model may be somewhat better than the non-linear model. As mentioned previously, the non-linear model may have the potential of applications where parameters set slightly outside the FFD ranges.

### Model Performance and Validation

The experimental values were found in good agreement with the predicted values from Eq. (2) to Eq. (3). Model performance was validated by the four key parameters at combinations of HPP/temperature/AITC concentration/time such as 280 MPa/-6°C/0.14%/16 min, 320 MPa/2 °C/0.12%/13 min, 340 MPa/-12°C/0.15%/12 min and 260 MPa/4 °C/0.15%/10 min (all within the full factorial design ranges). **Table 5** presented the log reduction (experimental vs. predicted), both models (Run #1, #2, #3, and #4) showed predictions with derivation within 10 and 25% for both the linear and non-linear models, respectively. The developed models were proven to be reasonably accurate for predicting the inactivation of *E. coli* O157:H7 in ground chicken meat with treatment parameters in the range of −15–4°C, 250–350 MPa, 0.05–0.15% and 10–20 min. In addition, we further evaluated the dimensionless non-linear model with some factors set outside design ranges, e.g., Run #5: 270 MPa/6 °C/0.2%/18 min, Run #6: 260 MPa/10°C/0.13%/25 min, Run #7: 380 MPa/2°C/0.2%/12 min and Run #8: 250 MPa/12°C/0.18%/15 min. The experimental vs. predicted values for Run #5/#6/#7/#8 values were 4.31/4.12/6.44/3.31 (experiment) and 4.95/4.53/6.57/3.59 (predicted by Eq. 3) log reduction of *E. coli* O157:H7, respectively, and all discrepancies were within 15% (**Table 5**).

**Table 5:** Verification of predictive models (Eq. 2 and Eq. 3) for log reduction of *E. coli* O157:H7 in raw ground chicken meat.

Run	Parameter				Log_10_ reduction (CFU/g)^a^		
	Temperature (°C)	Pressure (MPa)	AITC concentration (%)	Time (minute)		Log *N*_o_ – Log *N*	
					Experiment	Predict (Eq. 2)	Predict (Eq. 3)
1	−6	280	0.14	16	4.01 ± 0.44	4.28	4.97
2	2	320	0.12	13	4.11 ± 0.74	4.12	5.09
3	−12	340	0.15	12	5.79 ± 0.14	5.47	5.93
4	4	260	0.15	10	3.23 ± 0.46	3.19	3.07
5	6	270	0.2	18	4.31 ± 0.08		4.95
6	10	260	0.13	25	4.12 ± 0.41		4.53
7	2	380	0.2	12	6.44 ± 0.57		6.57
8	12	250	0.18	15	3.31 ± 0.16		3.59

*^a^Initial populations of E. coli O157:H7 was at 8.0 log CFU/g level. The detection limit was 1.0 log CFU/g.*

### *E. coli* O157:H7 Storage

**FIGURE 3:**
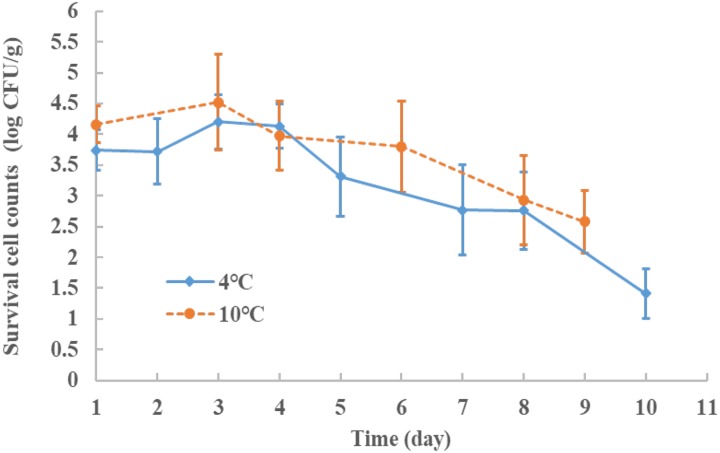
The survival behaviors of *E. coli* O157:H7 stored at 4 and 10°C, for 9–10 days after HPP treatment with conditions: 300 MPa, 15 min, 4°C and 0.12% AITC concentration (%). The initial inoculum counts of the *E. coli* O157:H7 was at 8.0 log CFU/g level.

Storage studies were conducted to determine the effect of the treatment parameters on *E. coli* O157:H7 survival. The storage test after treatment was performed in 7–10 day periods at 4°C (a typical refrigeration condition) and the cell counts were recorded at selected time interval. The results showed in **Figure 3**, with four factor conditions set at 0.12% AITC concentration, 300 MPa, 15 min at 4°C. After HPP processing, the samples were stored at 4°C for 10 days. The initial *E. coli* O157:H7 number without treatment was at 8 log CFU/g level. After the treatment at 1, 2, 3, 4, 5, 7, 8, and 10 days during the storage period, the survival *E. coli* O157:H7 were 3.74, 3.72, 4.2, 4.13, 3.31, 2.77, 2.76, and 1.41 log CFU/g, respectively. The cell population was slightly increased (by 0.5 log CFU/g at days 3 and 4) but still within the testing error, then, decreased to1.4 log CFU/g at day 10 and under the detecion limit at day 21 (<1.0 log CFU/g, datum not shown). It showed that AITC remained active and continously killed the damaged cells in this study. At abuse storage temperature, e.g., 10°C, a similar decreasing trend was observed but at a slightly slower rate and was under the detection limit at day 21. The *E. coli* survival counts were also tested on the non-selective media and results showed consistent trend having about 0.5 log CFU/g higher than selective 3M petrifilm (data not shown). Our ground chicken meat was treated with gamma irradiation to eliminate the background microbe noise which was tested/verified using TSA and non-selective petrifilm. Therefore, a proper combination of HPP and AITC may be able to deliver the 5-log CFU/g reduction during low temperature storage.

## Discussion

*Escherichia coli* O157:H7 survival behavior in response to HPP intervention included four parameters, i.e., hydrostatic pressure, process time, process temperature and AITC concentration, was investigated via an experimental design that further facilitated model development. High hydrostatic pressure (in terms of HPP) recently became a commercially feasible processing method to achieve the foodborne pathogens inactivation (Balasubramaniam et al., 2015) in certain foods thanks to the advancement in machinery design. However, raw meat texture and quality showed significantly visible change/damage in our study at 400–450 MPa which inspired the incorporation of natural food-grade essential oils (i.e., AITC) and, therefore, applying a lower hydrostatic pressure to increase microbial inactivation. Del Olmo et al. (2010) and Simonin et al. (2012) reviewed and reported that meat (poultry) quality was affected by high hydrostatic pressure at 400 MPa and higher pressure levels which is similar to our observations. Several studies also have reported that, in HPP alone experiments, meat quality (beef) was damaged at pressure level higher than 400–450 MPa (McArdle et al., 2010, 2011). To achieve optimal lethality (e.g., >5 log reduction) of *E. coli* O157:H7 through those complicated interactions it can only be attempted via a proper experiment design and mathematical analyses or model development. HPP alone may cause certain level of lethality for *E. coli* O157:H7, which was found in the range of 1–3 log CFU/g (300–400 MPa) in 10–15 min. The HPP alone (<400 MPa and <15 min) cannot achieve a 5 log CFU/g reduction of *E. coli* O157:H7.

Previous studies showed that combining the HPP (400–600 MPa) and temperature (−5 to 30°C) with different processing time (1–20 min) may inactivate 1–3 log of several *E. coli* strains in foods (Black et al., 2010; Omer et al., 2010; Baccus-Taylor et al., 2015). On the other hand, almost no literature focused on the impact of low HPP operation temperature (e.g., −15 to 5°C) on microbial survival to include foodborne *E. coli* O157:H7 in meat which may involve phase change (i.e., freezing/thawing) stages. The freezing/thawing of free water in food matrix under high hydrostatic pressure could be very complicated (e.g., phase change temperature might be shifted) and only a few publications (for meat) are available for this physical change phenomenon and its impact on microbial survival. We may assume that the phase change could occur in a fast path and ice crystal size might play some (unclear) role to cause cell structure damage – this should be a very interesting subject for further research. Since temperature and pressure are both important parameters and their impact including interaction is not well investigated yet, a proper experimental design was applied to develop the empirical model which may take into account the phase change effect on *E. coli* inactivation. Model development technology is a powerful method/tool in solving the complicated multi-parameter problem to achieve the challenging scientific/engineering result. Furthermore, the results also provides another possibility for commercial raw meat application, i.e., to choose an optimized temperature range in order to achieve the higher *E. coli* O157:H7 inactivations and reduce the after processing storage cost at or below refrigeration. Lethality affected by operation temperature may only appear in some food systems and AITC addition might further enhance the temperature effect.

Lu et al. (2016) showed that AITC had strong antibacterial property and high potential to effectively control *E. coli* O157:H7 in TSB when the concentration reached 1000 μg/ml. However, the AITC antibacterial efficiency did not cause significant difference when concentration was below 1000 μg/ml level. The antibacterial potentiality was found very weak without other hurdle used even when AITC concentration reached 0.25% (**Table 3**) which is much different from Lu et al. (2016) study. Our result indicates that the effect of AITC (or similar compounds) on foodborne pathogen inactivation should be carefully examined in actual targeted foods with selected experiment parameters/conditions and appropriate bacterial growth/recovery medium.

Hurdle technology is gaining in popularity in food processing in recent years due to its potential for extending the shelf-life and reducing negative change to food texture. For food safety concern to meet the ‘pasteurization’ status of non-thermal process such as HPP (National Advisory Committee on Microbiological Criteria for Food [NACMCF], 2006), HPP in couple with other processing means/aids to achieve a 5-log CFU/g reduction of foodborne pathogen may be required (Liu et al., 2012; Huang et al., 2013). Furthermore, the cost of HPP operation is affected by high pressure levels, holding time and temperature. It is highly desirable to optimize (minimize) the operation cost with food quality and microbial safety factors included (Bover-Cid et al., 2011). In our study, HPP alone (350 MPa, 4°C) in 15 min can only attain a 2.1 log CFU/g reduction of *E. coli* O157:H7 (**Figure 1**). Combining HPP at 350 MPa, 20 min at 4°C with 0.15% AITC concentration, a greater than 5 log reduction was obtained (**Table 4**).

High pressure processing may cause sublethal injury in bacteria (Wesche et al., 2009), where the cell membrane is a primary site of pressure damage (Yuste et al., 2004). The membrane damaged cells may show enhanced sensitivity to antimicrobials (Hauben et al., 1998). It has been reported that AITC can cause membrane damage which resulting the membrane became more permeable and increasing the leakage of cellular metabolites (Lin et al., 2000), the essential oil can also inhibit enzyme activities in *E. coli* O157:H7. Luciano and Holley (2009) reported three potential functions of AITC including (1) it is more effective at low pH and degradation reduces antimicrobial activity; (2) decomposition products in water lost antimicrobial function; (3) it may have multi-targeted action mechanisms – inhibiting several metabolic pathways and damaging cell structures.

Gänzle and Liu (2015) pointed out that some surviving cells (e.g., *E. coli* O157:H7) after HPP treatment may be able to repair the sublethal damage in suitable growth conditions to cause safety issue. In order to mitigate this concern with proving the concept that AITC surviving HPP may remain functional (i.e., active) to kill damaged cells, a storage test (up to 10 days) to observe how the cells would survive during 4 or 10°C was performed. **Figure 3** showed the survival counts of the *E. coli* O157:H7 were reduced to 3.50–4.20 log CFU/g after HPP, and could drop to 1.41 log CFU/g in 10 days at 4°C storage (typical refrigerated temperature). A similar trend was observed at 10°C for abuse temperature case. Based on the storage study results, pressure-damaged cells (*E. coli* O157:H7) in chicken meat after HPP+AITC treatment were found not able to recover. Therefore, combining the HPP and AITC, we found the hurdle effect on ground chicken meat can achieve an inactivation of *E. coli.* O157:H7 to >5 log CFU/g with lower pressure levels and AITC concentrations in this study. There could be some strains with higher resistance to pressure stress in which a 5 log CFU/g reduction may not be achieved.

Reliable mathematical models (linear and dimensionless non-linear) provide several advantages which include estimating the objective (i.e., cell count reduction) without doing experiment (another cost saving). Our models may also provide the useful means to locate/select the HPP operation conditions (i.e., <350 MPa) with a greater than 5 log CFU/g lethality achieved or implemented. Furthermore, the dimensionless non-linear model showed potential applications and reliable predictions with parameters slightly outside the designed limits (accuracy in 15% error vs. experiment data, **Table 5**, No. 5, 6, 7, and 8) which may expand its parameter application ranges. The users should apply those empirical models carefully to consider equipment, microbial strains, food matrix and other factors not to exceed those parameter boundaries. The model parameters may change as soon as a different type (or even batch) of meat is used. Until further validation, the model is valid only for the type of meat and equipment for which it was validated.

## Conclusion

The complicate interactions from four parameters which deliver complex and important impact on *E. coli* O157:H7 lethality in raw ground chicken meat under HPP stress can be expressed by the linear regression and/or dimensionless non-linear models (Sheen’s model). The identified key parameters include pressure (250–350 MPa), operation temperature (−15–4°C), AITC concentration (0.05–0.15%, w/w), and pressure-holding time (10–20 min), where the pressure level is lower than those needed and used in the meat industry (>450 MPa) to achieve a 5-log CFU/g lethality. The reduced pressure level (≤350 MPa) may also play an important role in effectively reducing the meat quality and/or texture damage. With the current developed method, meat quality may be much improved and potential operation cost reduced. The HPP process optimization is also feasible via model application.

## Author Contributions

C-YH completed the experiment, data analyses, and initial writeup. SS supported lab facility, tools, advice and submitted the manuscript. CS provided information, uropathogenic *E. coli* strains, and feedback. L-YS provided general advice to student C-YH.

## Disclaimer

Mention of trade names or commercial products in this publication is solely for the purpose of providing specific information and does not imply recommendation or endorsement by the United States Department of Agriculture. USDA is an equal opportunity provider and employer.

## Conflict of Interest Statement

The authors declare that the research was conducted in the absence of any commercial or financial relationships that could be construed as a potential conflict of interest.
